# A practical approach to orofacial rehabilitation in a patient after inferior maxillectomy and rhinectomy with mono framework construction supported on a zygomatic implant placed in the glabella: a case report

**DOI:** 10.1186/s40902-021-00312-8

**Published:** 2021-07-13

**Authors:** Vivek Gaur, S. Mahendra Perumal, Faizur Rahmaan, Łukasz Pałka

**Affiliations:** 1 Department of Oro-Maxillofacial Surgery, Jaipur Dental College, Jaipur, India; 2 Ghaziabad, India; 3 Department of Oral and Maxillofacial Surgery, K S R Institute of Dental Science and Research, Erode, Tamilnadu India; 4 Cura Dental Foundation, Channai, India; 5 Private Dental Practice, Zary, Poland

**Keywords:** Single-piece implants, Glabella, Zygomatic implants, Pterygoid implants, Nasal epithesis

## Abstract

**Background:**

In the field of craniofacial tumor surgery, an adequately performed excision, despite being a life-saving procedure, is only a first step to successful treatment. During such a procedure, the main goal is to completely remove the lesion, paying less attention to factors contributing to future rehabilitation possibilities. One ty 2of the possibilities for prosthetic rehabilitation of such cases is utilizing one-piece implants with bicortical anchorage.

**Case presentation:**

This case report presents a case of a 48-year-old patient with oral squamous cell carcinoma (OSCC). The treatment protocol consisted of radical surgery to remove the tumor, and intraoral and extraoral rehabilitation with a single framework prosthesis anchored with one-piece implants. Moreover, the intraoral stomatognathic deformity was corrected with a fixed implant-retained prosthesis, and the extraoral defect was covered with a removable epithesis.

**Conclusions:**

The use of one-piece implants with bicortical anchorage may be an additional tool in reconstructing maxillofacial defects. Properly executed treatment may improve the esthetics, speech, masticatory function, muscle support, and the overall quality of life of patients with extensive defects in the maxillofacial region.

## Background

According to Borle, “there is no scope of conservative surgery while treating the malignancy, and the first chance is the best chance for the surgical resection”—these are the aims each operator pursues when treating oncology patients [1]. In maxillofacial surgery, when the lesion is localized in the maxillary region, resection of the structures integral for phonetics, deglutition, and mastication function must be performed since it is necessary for the patient’s survival. When the esthetically significant structures like ear, nose, and orbit are involved in the neoplastic process, there is also a need to address the severe emotional burden the patients will have to bear not only during the surgery but during rehabilitation as well. Thus, optimal esthetical and functional reconstruction and rehabilitation of the ablative structures are necessary for the social integration, psychosomatic, functional, and the quality of life (QOL) of patients undergoing such extreme surgical procedures [2–4].

To achieve success in all these fields, surgeons should closely cooperate with prosthetic specialists like anaplastologists [5]. Their main aim is to restore a practical division between oral, nasal, or orbital cavities and cover the ablative defect, usually by vascularized free tissue transfer (VFTT) [6]. Nevertheless, there are limitations of the flap reconstruction regarding its availability and mobilization to cover up the extent of the defect. What is more, there are several complications with flap advancement, including infections, bleeding, and displacement of free margins [7].

The defects which could not be covered are restored with a craniofacial prosthesis or epithesis. The main advantages of epithesis include minimal or no surgical procedure and restoring the esthetics and function in a near-natural appearance with immediate results. Placing a nasal epithesis is a demanding procedure, but also promising when compared to surgical options as a nasal septal perforation is through-and-through defect with no underlying mucoperichondrium or mucoperiosteum [8, 9].

In this paper, the authors present a concept where the patient’s intraoral and extraoral defect was rehabilitated by a single framework as a fixed solution for intraoral stomatognathic defect and extraoral removable epithesis. Achieving tripodization in the maxilla was possible with long one-piece implants with distant cortical anchorage.

## Case presentation

**Fig. 1:**
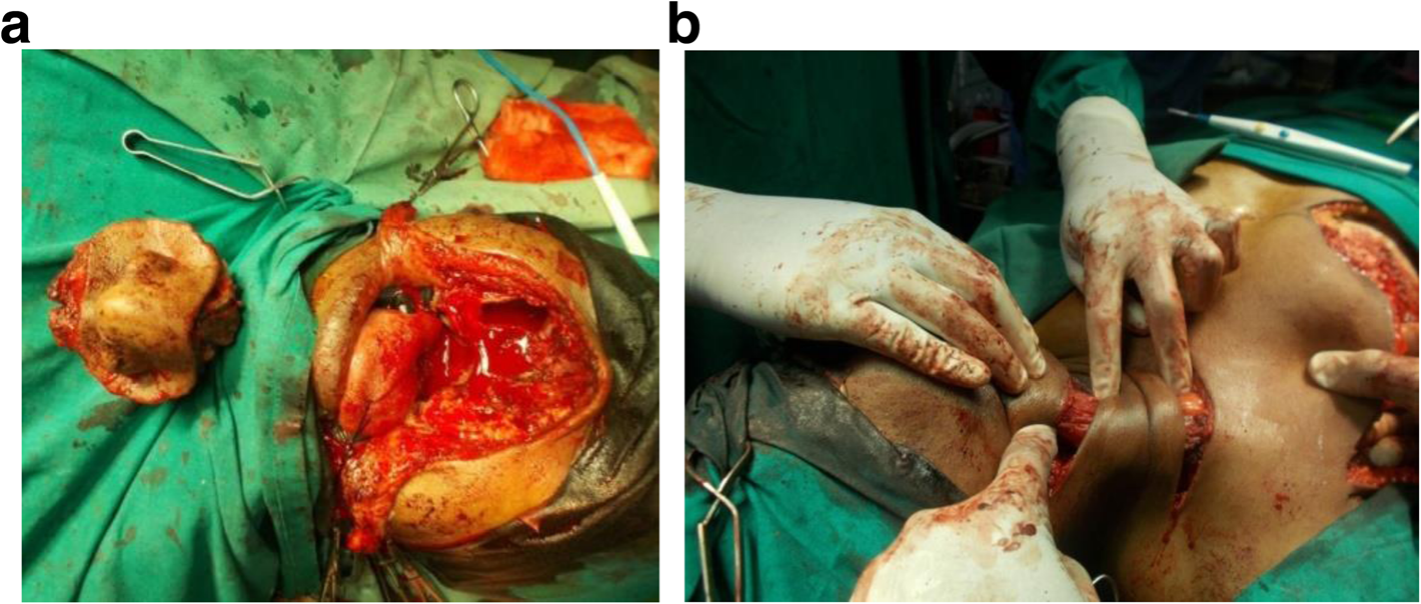
**a** The procedure of rhinectomy and right antrostomy. The anterior maxillectomy with disease-free margins covering the nasal area, right maxillary sinus, and the pyriform aperture till the ethmoid bilaterally. **b** The procedure of a flap tunneling up to the defect as planned through the neck and masseter. The mobilization of pectoralis major myocutaneous flap tunneling behind the sternocleidomastoid muscle and platysma

A 48-year-old man was referred to the maxillofacial clinic of Mannan Hospital, Chennai, India, with a severe face deformation and difficulty in chewing with restricted mouth opening. He has undergone anterior segmental maxillectomy along with right antrostomy and complete rhinectomy 4 years back as a treatment for oral squamous cell carcinoma, as shown in Figs. 1a and 3a.

The post-surgical defect was closed after mobilizing the pectoralis major myocutaneous flap (PMMC) with advancement and bipedicling (Fig. 1b) with flap shift to the operation site by modified McFee incision and supra masseteric tunneling. The skin between the two pedicles was denuded and separated into two layers—the first layer was sutured to perinasal skin, and the latter one to the palatal region covering the intraoral and extraoral defect as tightly as possible, resulting in nasal defect coverage and obliteration of oro-antral communication. After the surgery patient did not undergo any additional radio- or chemotherapy.

**Fig. 2:**
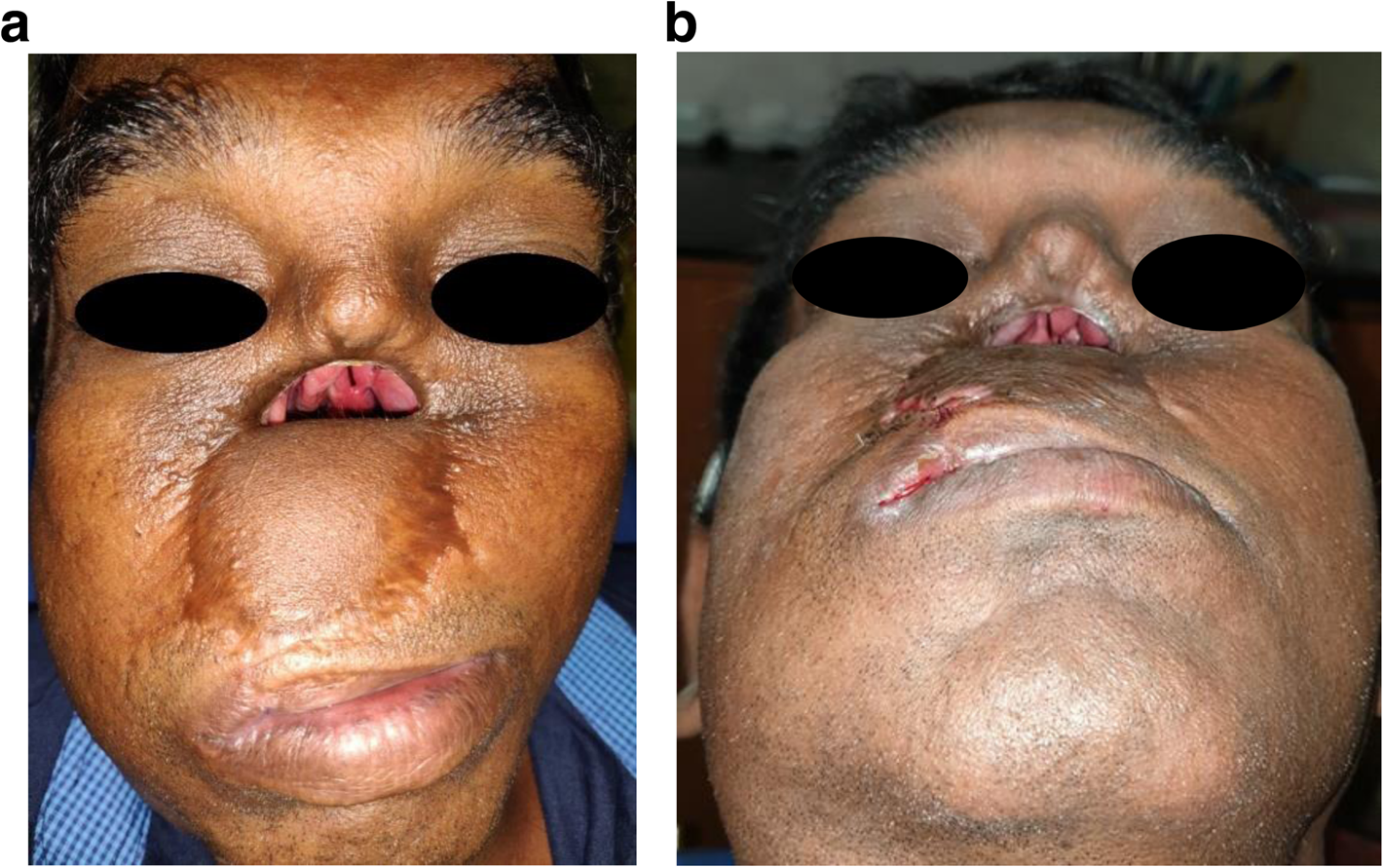
**a**, **b** Pre-operative facial view—a broad nasal opening is visible, which led to anterior facial deformity

At the first visit, the patient’s intraoral and extraoral conditions were examined, i.e., the remaining teeth and lost stomatognathic structure were evaluated. Based on Brown’s classification, the defect had been assessed as class 2b, with a vertical and horizontal component [10]. During the physical examination, a broad nasal opening was found, which led to anterior facial deformity (Fig. 2). Moreover, hyper-nasal tone, abnormal breathing pattern, inability to chew, masticate, and speak properly were revealed and the upper lip was short with minimal mobility.

Intraorally, all the upper incisors up to premolars were missing bilaterally, as well as lower left molars. Prior to implant placement, all the remaining teeth were extracted due to their questionable periodontal support. There was excess bulk of the PMMC flap on the right side and restricted mouth opening and constricted right corner of the mouth. Furthermore, the upper lip construction was deficient in loose tissue lacking the spread and resiliency needed for the normal contour, such as lip commissure, columns, and nasolabial fold.

Extraorally, as of the bulky skin island and the flap, the base for the nasal epithesis was devoid of supporting nasal and alveolar bone. Anteriorly, the resection included septum, the nasal piriformis, and support of the alar base. Because of the patient’s fear of additional surgeries, hard tissue augmentations like fibula or iliac crest bone graft were not performed. One of the consequences of this decision was the lack of stable bone in the front of the maxillae for the implant placement.

To solve these problems, additional zygomatic implant engaging glabella/floor of the frontal sinus was chosen in order to anchor the implant in the native bone which, together with the pterygoid implants, would allow to create enough support for the implant-retained prosthesis with nasal epithesis. This solution was offered to the patient as one of the possible treatment options, which the patient readily accepted and agreed to.

**Fig. 3:**
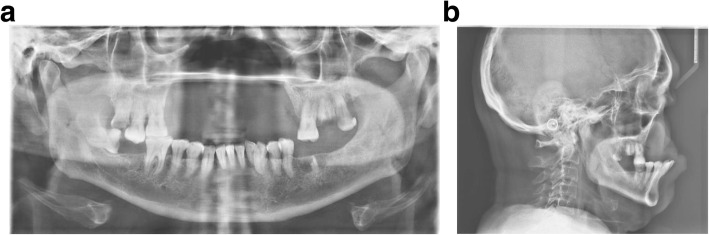
**a** Pre-operative OPG. All the upper incisors up to premolars missing bilaterally, as well as lower left molars. **b** Pre-operative laterocephalogram

Cephalometric analysis and computer tomography were performed (Fig. 3a and b) to evaluate the facial profile and to look for possibilities for anterior implant anchorage in order to achieve a respectful facial profile, most favorable loading conditions, and functionality of the future prosthesis.

**Fig. 4:**
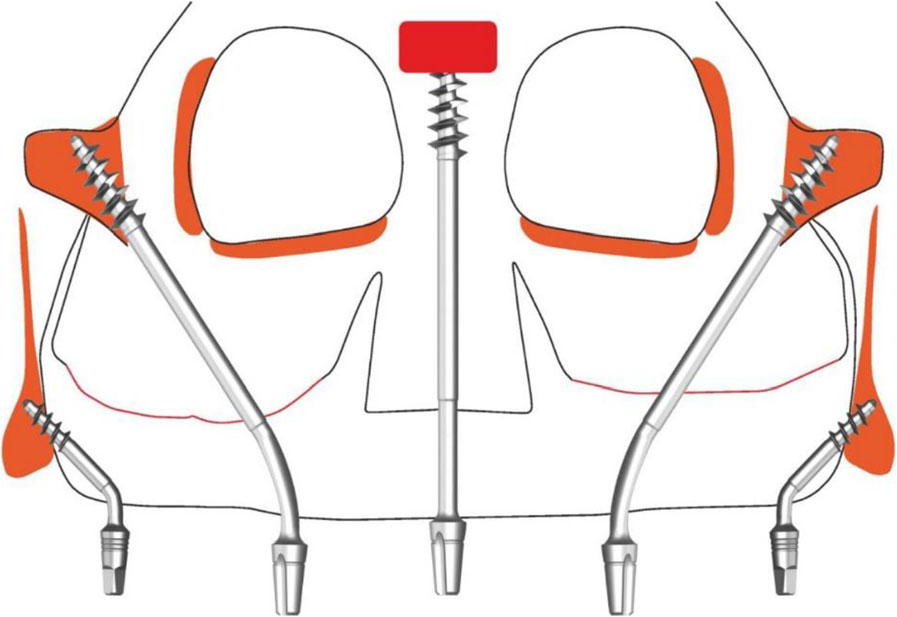
Schematic planning of the implant placement

**Fig. 5:**
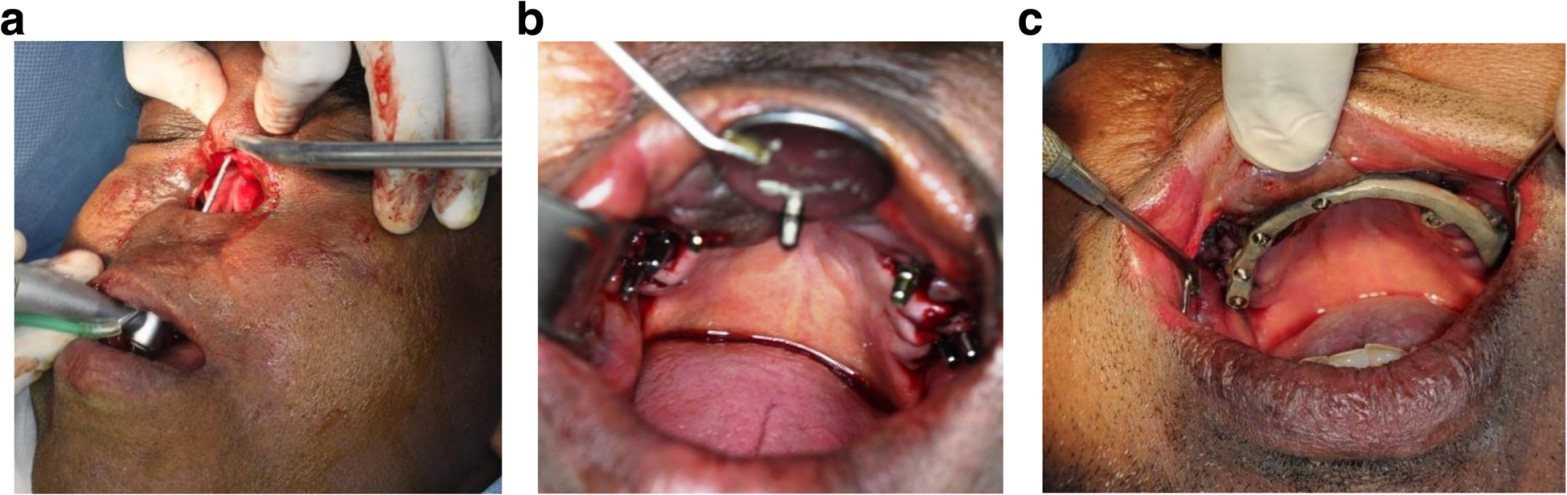
**a** Glabella implant osteotomy. **b** Implant placement procedure. **c** Metal try-in

**Fig. 6:**
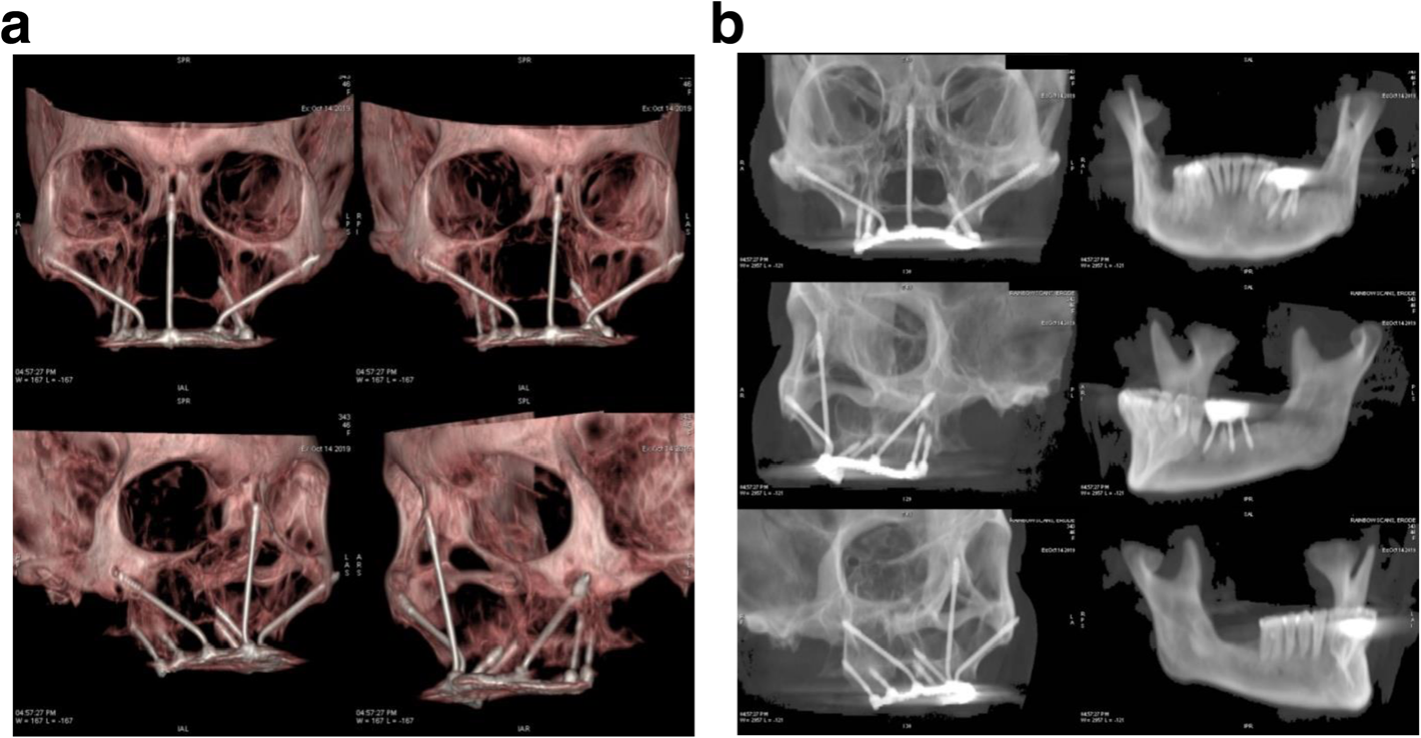
**a** Post-operative CT coronal view: one zygomatic implant was used for the glabella region anchorage, engaging the floor of the frontal sinus, and in the mandible, three implants were placed in order to replace the left molar. **b** Post-operative CT sagittal view

Under local infiltration, the implants were placed in a flapless procedure, successfully engaging pterygoid apophysis and zygomatic bone bilaterally in the maxilla and achieving high primary stability > 70 Ncm as listed in Table 1 (Figs. 4, 5b, and 6a). Additionally, one zygomatic implant was used for the glabella region anchorage, engaging the floor of the frontal sinus [6]. The osteotomy for glabella was made keeping the surgically compromised upper lip in line with nasion for the future emergence profile of the glabella abutment, and implant osteotomy was made through the lip toward between the inner canthus of the eyes holding the remaining nasal bridge by fingers to perform a flapless procedure.

**Table 1:** Types of implants inserted

Type of implant	Location	Length	Diameter	Number of implants
BECES (Simpladent, Switzerland)	Pterygoid	26 mm	3.6 mm	1
BECES (Simpladent, Switzerland)	The pterygomaxillary buttress on the right side	17 mm	3.6 mm	1
BECES (Simpladent, Switzerland)	Pterygoid (distal)	17 mm	3.6 mm	1
BECES (Simpladent, Switzerland)	Pterygoid of the left maxilla	23mm	3.6 mm	1
ZDI (Simpladent, Switzerland)	Zygomatic bone on the right side	50 mm	4.6mm	1
ZDI (Simpladent, Switzerland)	Zygomatic bone on the right side	52.5 mm	4.6 mm	1
ZDI (Simpladent, Switzerland)	Glabella region engaging floor of frontal sinus/nasion; the fusion of middle and superior transverse facial buttress	55 mm	4.6 mm	1

In the mandible, three implants were placed in order to replace the left molar (Table 1) (Fig. 6b). Jaw relation recordings were made with bite registration wax after anthropometric VD measurements, following the “equal thirds concept” [11].

**Fig. 7:**
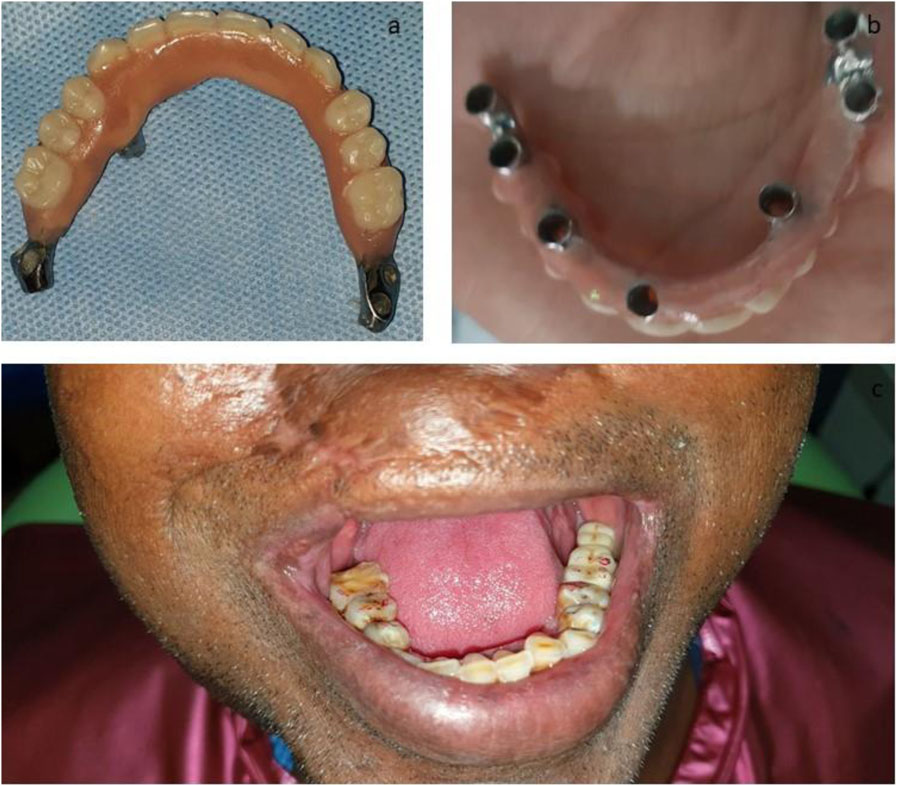
**a**-**c** The metal to acrylic hybrid prosthesis. The anteriors were set up in a crossbite and the posterior teeth accordingly to the concept of strategic occlusion

It had been decided to reconstruct the maxillary defect with metal to acrylic (Ivoclar Vivadent AG) hybrid prosthesis (Fig. 7a-c). The anteriors were set up in a crossbite and the posterior teeth accordingly to the concept of strategic occlusion. In this type of occlusion, non or semi-anatomical teeth are used with lingualized occlusal contact points kept only on the medial part of the first molar and both premolars [12].

The next day, the metal framework was fitted on the implant abutments and, after adjustment, permanently cemented with the use of glass-ionomer cement (Fuji Plus; GC). Bilateral balanced occlusion with single point contact on the fossa of lower teeth and the slightly anterior crossbite was chosen occlusal scheme as previously mentioned.

**Fig. 8:**
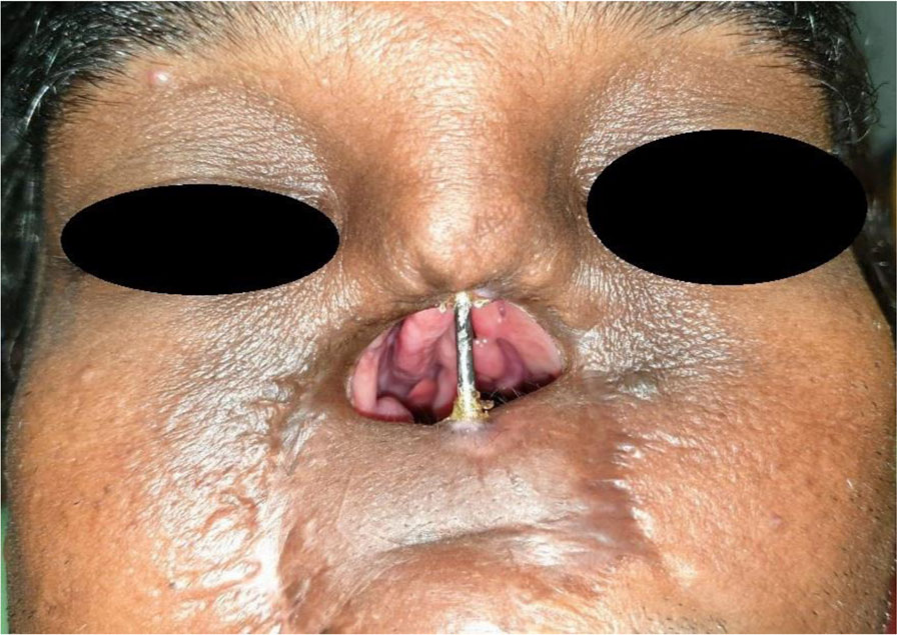
A post-surgery view of the zygomatic implant placed for the glabella region anchorage after the intraoral prosthesis fixation

**Fig. 9:**
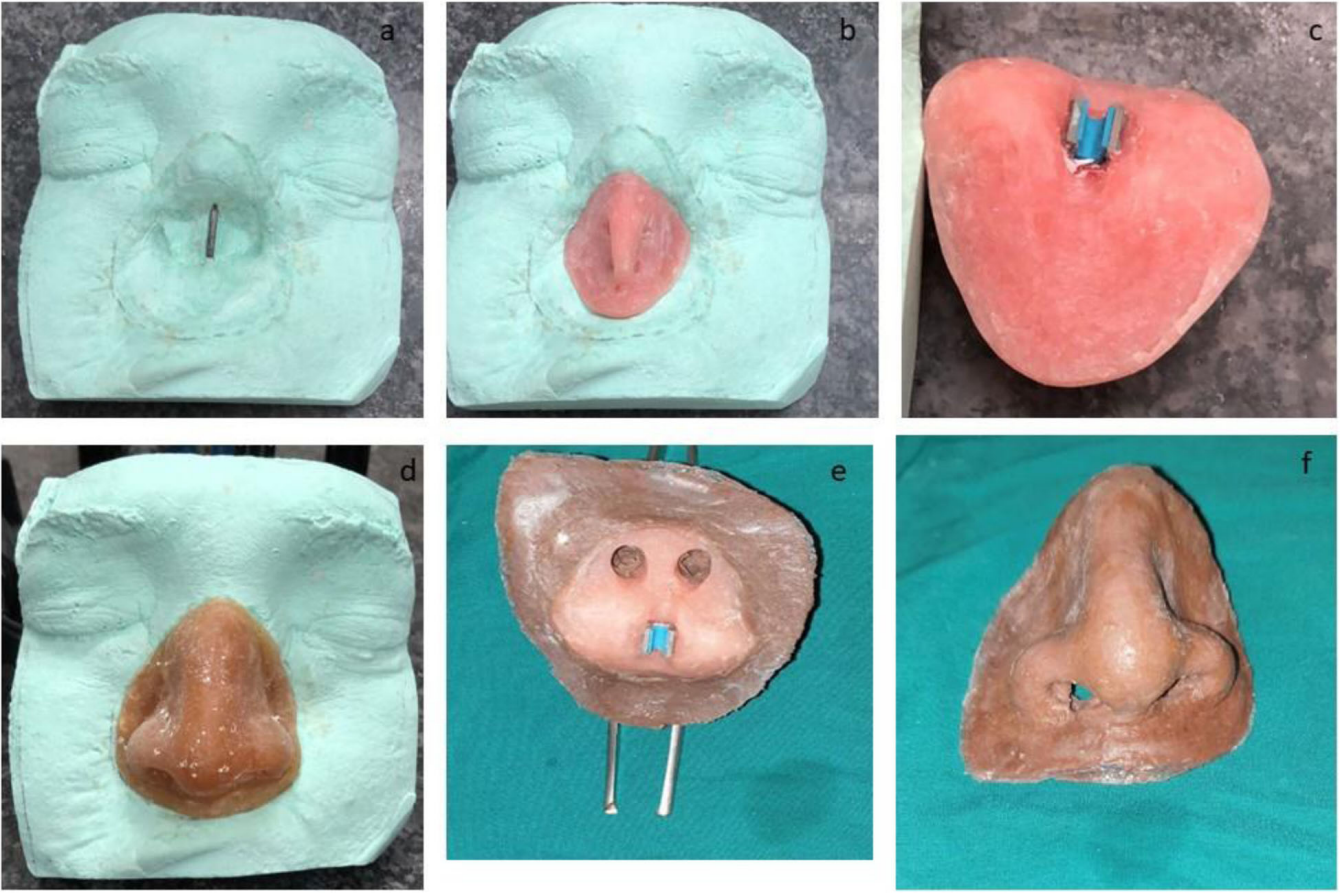
A removable nasal epithesis with a custom made bar clip. **a**-**f** Steps of making the nasal epithesis. The clip was fabricated by doing casting of wax pattern over the body of the zygoma implant; then, it was cut in half. Retentive wings were also fabricated along the clip. The half-cut clip was fixed to the tissue surface of the nasal epithesis to be used as a clip over the body of zygoma as a bar

For the nasal epithesis fabrication, we have chosen the silicone material (Technovent) because of its excellent compatibility with the soft tissue, color, and pigmentation reproduction and blending with the native tissue margins (Figs. 8 and 9) [13]. The clip was fabricated by doing casting of wax pattern over the body of the zygoma implant; then, it was cut in half. Retentive wings were also fabricated along the clip. The half-cut clip was fixed to the tissue surface of the nasal epithesis to be used as a clip over the body of zygoma as bar.

**Fig. 10:**
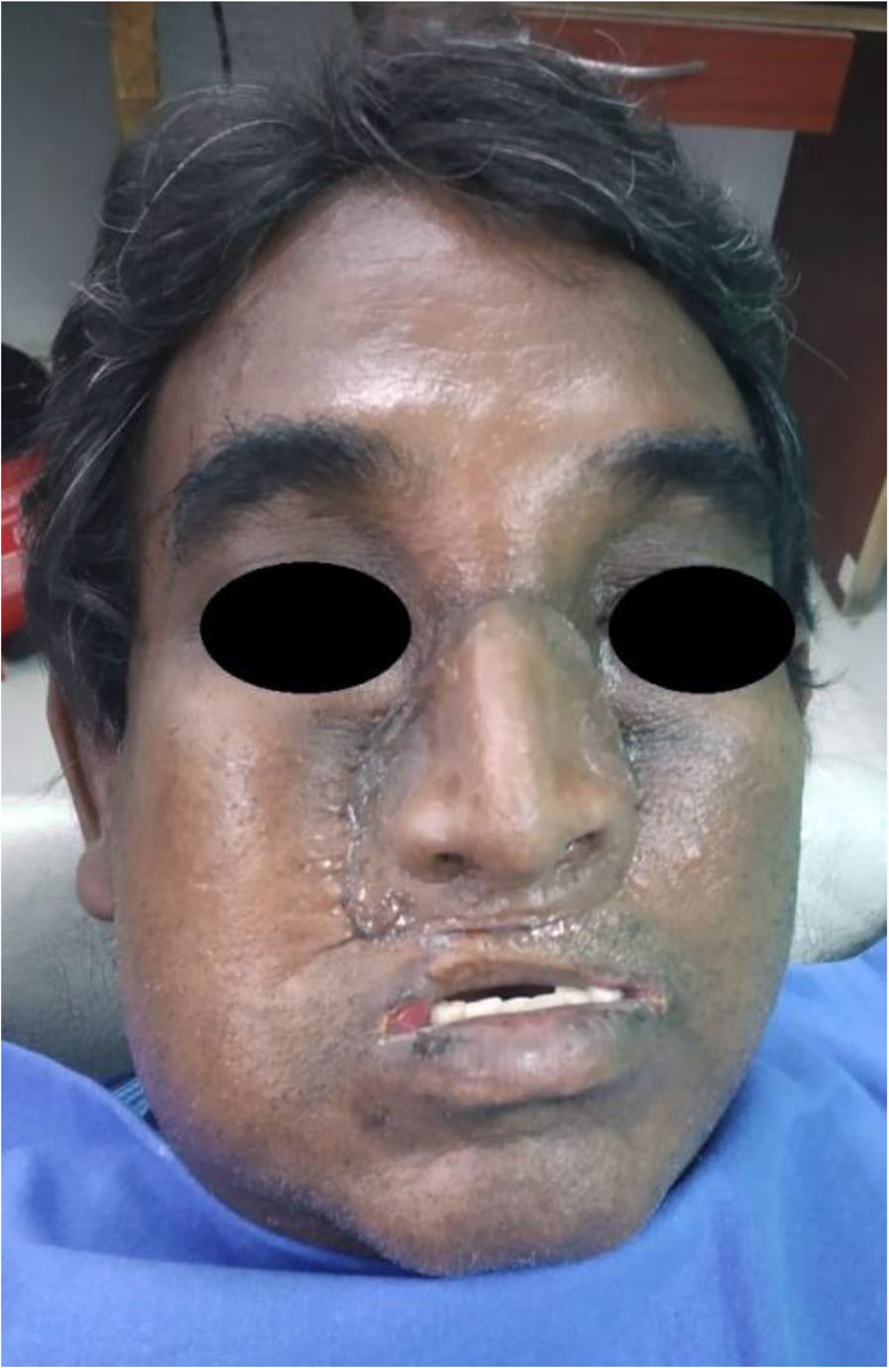
Final look with an intraoral hybrid and extra oral-nasal epithesis

For the retention of the nasal epithesis, we adopted a unique, self-developed concept—a maxillofacial prosthodontist fabricated a clip with the diameter of the body of the ZDI (Simpladent) implant, which was used as a bar and clip attachment, with the bar used as the anterior ZDI (Simpladent) implant body (Figs. 9 and 10). It is a removable epithesis fabricated with washable silicon material for home care by the patient. The patient was instructed regarding hygiene and maintenance of the prosthesis.

**Fig. 11:**
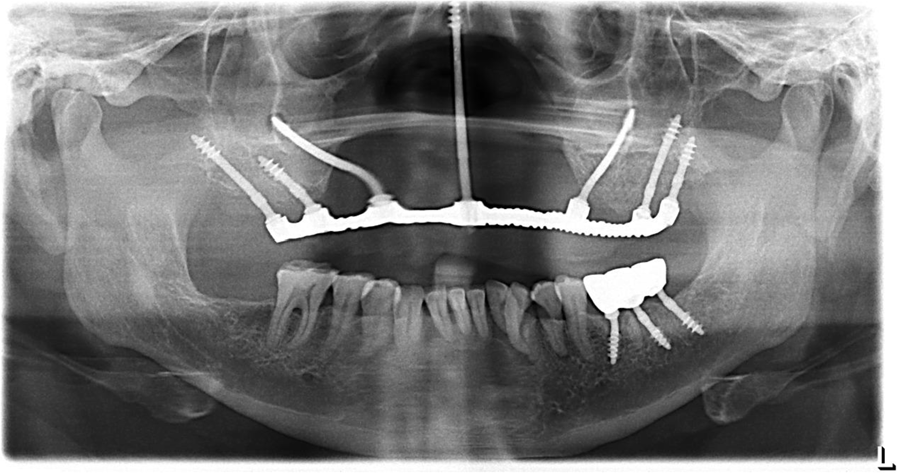
One-year follow-up OPG

At 1-year follow-up visit, the patients did not report any complications (Fig. 11).

## Discussion

Rehabilitating resection cases with dental implants is a tedious and challenging task. The purpose of the study was to present the possibilities of implant treatment in the case of severe hard and soft tissue deficiencies, achieve tripodization resisting offset forces in the maxilla for the long-term successful prosthetic rehabilitation and survival, and the use of these implants for epithesis retention.

Classic endosseous implants are mostly conical root forms having full crestal two-piece design with a tapered apical area with a rough surface body, which becomes a nidus for future bacterial accommodation [14]. In the case of single-piece implants, the occlusal load is distributed through apically engaged threads, which are splinted, and their design is like the Toulouse leg screw with different core and thread diameters [15]. Since they are single piece with a smooth surface, they have the most acceptable biological seal at the mucosal or skin level [16]. Zygomatic bone engagement is one of the most evidence-based techniques when restoring the resected or atrophic cases [17–21]. On the other hand, conventional zygomatic implants have a wide rough surface at the crestal cortical, leading to peri-implantitis, fenestrations, oro-antral communication, palatal emergence resulting in low patient satisfaction, and speech impairment [22].

In the presented case, zygomatic implants function as an extra-maxillary implant following conventional zygomatic anatomy-guided approach (ZAGA) classification, transmitting all the masticatory forces at the zygomatic buttress [23]. Because of the bending zone, the abutment can be moved to a desirable prosthetic position irrespective of the angle of the placement to the zygomatic buttress. Thus, it achieved an outcome of a minimal or low palatal emergence and patient’s satisfaction. ZDI implants are suitable for offset loading but not against axial loading; therefore, when applied, they need to be splinted with the anterior or posterior rigid implants. This widens the supporting polygon enhancing the prognosis of the construction against the lateral masticatory forces [24–26].

One of the principles of single-piece implants placement is to place them in different directions and at different angles; thus, when splinted, they would resist the lateral forces [27].

Different methods for the epithesis anchorage are present, including anatomical, chemical, mechanical, and surgical. Among these types, implant-retained epitheses are most advantageous as we can achieve optimal camouflage with the desired esthetical result [28]. Bone anchorage with implants has enhanced and reliable retention not affected by external environmental factors like sweating and is convenient to wear by the patient. Most studies present the construction of the nasal craniofacial prosthesis framework by zygomatic and small glabella implants combined with the support from the piriform aperture engaging the nasal floor or the nasomaxillary buttress. In this case, there was no nasal base and the lateral wall bone which could be used for implant anchorage. Thus, the prosthetic idea was to make margins over the mobile soft tissue. In the case of complete rhinectomy, a prosthodontist can create an epithesis as a single unit [29]. Silicon material was preferred for the nasal epithesis as hair and pigmentation can be embedded easily. It is light and very tissue friendly and has feather margins to mask over the recipient site. Different options are available for the retention over the implant-anchored framework like bar and clip, Dolder system, magnet system Magan-Cap System. Still, in the presented case, we decided on the most cost-effective and practical approach by making a retention clip for the bar over the zygomatic implant (ZDI) body. The clip was custom-made from the body of the zygomatic implant.

Nasal epithesis designing and fabrication were performed according to recommended guidelines based on frontal face analysis [1].

Main limitation of this type of treatment is difficulty in zygomatic, tubero-pterygoid, and glabella osteotomy and implant placement especially without anatomical landmarks. Also, bending the implants requires a lot of training and experience to avoid bone fracture or loss of implant stabilization. Immediate loading puts a lot of pressure on the dental laboratory which needs to fabricate the complete prosthetic work in only few days.

To the best of our knowledge, this is the first case with the one-piece zygomatic type implant used for the frontal sinus floor anchorage. Also, the type of implants, tripodization achieved in the maxilla, which allows immediate functional loading, fixed intraoral prosthesis, and removable epithesis from single framework construction, have not been previously described in the literature.

## Conclusions

The successful use of one-piece implants with bicortical anchorage described in this report gives operators an additional tool for maxillofacial reconstruction. The use of frontal sinus as a distant bone anchorage can be used for epithesis and intra-oral prosthesis retention at the same time. Properly executed treatment improved the esthetics, speech, masticatory function, muscle support, and overall quality of life.

## Data Availability

Data sharing is not applicable to this article since no dataset was generated or analyzed during the current study.

## Abbreviations

OSCC: Oral squamous cell carcinoma
QOL: Quality of life
VFTT: Vascularized free tissue transfer
PMMC: Pectoralis major myocutaneous flap
ZAGA: Zygomatic anatomy-guided approach
